# Study on the temporal and spatial relationship between public health events and the development of air transport scale: A case of the Southwest China

**DOI:** 10.1371/journal.pone.0301461

**Published:** 2024-04-09

**Authors:** Zihan Li, Xiwen Deng, Yi Mao, Jinglong Duan

**Affiliations:** Department of geography, Shandong Normal University, Jinan, Shandong, China; Libyan Academy, LIBYA

## Abstract

The spread of the COVID-19 had profoundly affected the development of the air transportation. In order to determine the changes in air transportation volume associated with the development of the epidemic, this paper takes Southwest China as the study area. Monthly data and methods, such as the coefficient of variation, rank-size analysis and spatial matching index, were applied. The results found that: (1) during 2020–2022, there was a positive relationship between passenger volume and epidemic development, while freight volume increased for most airports in the first quarter of 2020–2022, particularly in the eastern region; (2) From the perspective of changes in air transportation volume under the development of the COVID-19, among various types of airports, the changes in transportation volume of main trunk airports were more significant than those of regional feeder airports in remote areas; (3) however, under the influence of the epidemic, main trunk airports still exhibited stronger attraction in passenger volume. That is to say, the passengers who chose to travel by air still tended to choose the main trunk airports and formed the agglomeration distribution pattern which around high-level airports in the provincial capital. Whereas the freight volume had a tendency of equalization among airports in Southwest China; (4) Over the course of time, the consistency of the spatial distribution of the number of cases and the passenger or freight volume in southwest China gradually increased. Among them, the spatial matching rate of the passenger volume and the number of COVID-19 cases was always higher than that of the cases and freight volume, which might indicate that there was a stronger correlation relationship. Therefore, it is proposed that the construction of multi-center airport system should be strengthened, the resilience of the route network for passenger transportation should be moderately enhanced, and the risk-resistant capacity of mainline airports and airports in tourist cities should be upgraded, so as to provide references for the orderly recovery of civil aviation and regional development.

## Introduction

From the end of 2019, the COVID-19 pandemic had significantly impacted all facets of human activity and socioeconomic operations. This caused a widespread concern from various fields across various nations or regions [1–4]. A multitude of studies had illustrated that population structure, factors mobility, regional connectivity network [5, 6], urban hierarchy [7], economic resilience and robustness [8, 9], digitalization level and development [10, 11], as well as the built environment [12, 13], all of them were closely related to and interact with the spread of the COVID-19 pandemic. Especially, there are many studies proving that regions with high population density and mobility tended to show a higher difficulty in controlling the epidemic [14–16]. Therefore, controlling population mobility had once become an essential and widely adopted countermeasure [17, 18]. Building on this, numerous scholars have explored the impacts on transportation and resulting changes.

It has been shown that the correlation relationship between COVID-19 cases growth and medium-distance travel is most obvious [19], which has dramatically changed people’s travel frequency and transportation mode [20]. The travel frequency and transportation mode of residents are obviously affected by the suspected/confirmed cases in local and nearby areas, with a noticeable increase in walking and private car travel. However, there are differences among different provinces, for example, the travel of Xizang and Qinghai is relatively less affected by the epidemic, while Hubei province has the biggest change compared with the non-epidemic period. Under the background of epidemic prevention and control, railway transport was more affected in cities with higher network centrality, while there is no fundamental change on the overall connectivity pattern of the national railroad network [21]. In the Guangdong-Hong Kong-Macao Greater Bay Area, passenger volume dropped significantly under Level I response to public health emergencies, while freight volume did not change significantly [22]. With the phased evolution of the epidemic, existing studies had found that under the "Stay local for the Spring Festival" initiative, the passenger volume of the Spring Festival in 2021 has decreased significantly but recovered faster than that in 2020. This indicates that the prevention and control policy has reduced travel, but its impact is significantly less than the epidemic itself [23]. In addition, some scholars also proposed suggestions such as optimizing transportation organization, introducing operating subsidies, and paying attention to passenger flow dynamics in different cities and at different stations [24].

In addition to ground transportation, the impact of the epidemic on air transportation is also prominent. Many studies had explored the connectivity and robustness of the air transport network [25, 26]. By comparing the connectivity of nearly 8,000 cities around the world, studies found a quite heterogeneous spatial recovery pattern, and whether the flight ban was implemented in time also had a great impact on dealing with the mutated virus [27, 28]. Due to restrictive bans on international travel and airline operations, the Asia Pacific has been more affected than the United States and the Europe [29]. The number of international flights and airlines in China dropped significantly. Besides, the connectivity efficiency of the aviation network was significantly reduced and characterized by spatial heterogeneity. However, the spatial pattern did not change fundamentally. For example, the agglomeration of the three major gateway airports in China was improved during the epidemic period [26]. However, the relevant discussions are not rich enough, especially the integration discussion and detailed comparison of passenger and freight transport volume in different periods before and during the comprehensive epidemic, which deserves further attention.

Therefore, taking Southwest China as an example, this paper uses spatial analysis, rank-size analysis, as well as proposed the improved spatial matching index, to explore the monthly changes in air transportation volume under the impact of the epidemic and the spatiotemporal relationship characteristics from 2020 to 2022. This paper aims to further enrich the empirical evidence on the transportation geography under the public health events. By exploring the relationship, we hope to provide reference for the recovery of related industries and better response to other possible emergencies in the future.

## Study area and its airport construction

### Overview of the Southwest China

Southwest China covers 5 provincial-level administrative units, including Chongqing, Sichuan, Guizhou, Yunnan, Xizang Autonomous Region (abbreviated as Xizang), with a total area of 2.34 million km^*2*^. This region spans the first and second terrain steps of China, involving the Qingzang Plateau, the Hengduan Mountains, the Yunnan-Guizhou Plateau, the Sichuan Basin and many other topographic areas. Except the complex and varied terrain, as well as the changeable climate, this region also has the different types of social and economic development. Not only are there regional central cities such as Chongqing and Chengdu, but also many remote or plateau mountainous areas with relatively lagging development. Besides, the Southwest China has rich tourism resources, making it an important tourist destination in China. By the end of 2021, the population of the Southwest China is about 200 million, but the overall level of economic development and urbanization rate are slightly lower than the national average.

### The air transport system construction in Southwest China

As of 2023, 54 civil aviation licensed transportation airports had been built in the Southwest China. Among them, 15 airports in Sichuan Province, such as Chengdu Shuangliu International Airport and Chengdu Tianfu International Airport; 15 airports in Yunnan Province, such as Kunming Changshui International Airport; 11 airports in Guizhou Province, such as Guiyang Longdong International Airport; 6 airports in Xizang, such as Lhasa Gongga International Airport; and 5 airports in Chongqing Municipality, such as Chongqing Jiangbei International Airport. Among them, Chengdu Shuangliu International Airport, Chongqing Jiangbei International Airport and Kunming Changshui International Airport are the main regional hub airports in China, and play an important role in China’s civil aviation system. Besides, in terms of airfield area class, there are four 4F class flight areas, two 4E class flight areas, nine 4D class flight areas and the remaining 39 airports in the region are 4C class flight areas.

## Methods and data

### Methods

(1) Coefficient of variation. The coefficient of variation was used to evaluate the variability of the COVID-19 cases and the air transportation scale of each city and airports in the Southwest China. Referring to the existing studies, the coefficient of variation is the ratio of standard deviation to the mean value. For its result, the higher the value, the greater the disparity among different study units [30].
(2) Rank-size analysis. This paper carried out the rank-size analysis on the basis of fractal theory to make further exploration on the structural characteristic. COVID-19 cases and the passenger and freight volume of air transportation, as well as their changes, were used. The formula as Eq (1) [31, 32]:
$$\mathrm{lg}P_{i}=K-q\cdot \mathrm{lg}(R_{i})$$ (1)
  where, *P* represents the scale, and *R* represents the rank order. *K* is the constant, while *q* is used to reflect the degree of concentration of the indexes. *q*>1 indicates that the major regions or indexes are prominent and concentrated in distribution, and vice versa for relative balance.
(3) Global spatial auto-correlation. In this paper, Moran’s I was used to determine the agglomeration level of elements’ spatial distribution pattern in the whole region, which is calculated as in Eq (2):
$$I=\frac{n\cdot \sum_{i}^{n}\sum_{j\neq i}^{n}w_{ij}(X_{i}-\bar{X})(X_{k}-\bar{X})}{\sum_{i}^{n}\sum_{j\neq i}^{n}w_{ij}\sum_{i}^{n}{\left(X_{i}-\bar{X}\right)}^{2}}$$ (2)
  where *w* is the spatial weight, *X* is the attribute value such as epidemic or airport traffic, *i* and *j* indicate different sub-regions, *I* value is between [–1,1], *I* is greater than 0 indicates that the spatial distribution has a positive correlation, i.e., the distribution of the attribute value is more neighborly similar to the regularity, and vice versa is negative correlation, and equals to 0 is completely irrelevant.
(4) Pearson correlation coefficient. The Pearson correlation coefficient was used to measure the correlation between different variables. The correlation results are characterized by *r*. The larger the |*r*| is, the stronger the correlation between variables. Besides, *r*>0 indicates a positive correlation between variables, while *r*<0 indicates a negative correlation between variables.
(5) Standard deviation ellipse and Spatial matching index. The standard deviation ellipse reflects the spatial dispersion of the elements, the range of one standard deviation covers 60% of the spatial content, small area indicates concentrated distribution, and the center of its circle is the spatial center of gravity measured by the elements and their indicators [31]. On this basis and the analytical idea of spatial overlap index and spatial misalignment index [33], this paper utilizes the intersection rate of the standard deviation ellipses and the distance between centers of gravity to construct the spatial matching index:

$$SC=n\sqrt{\frac{S_{\left(S1\cap S2\right)}/S_{\left(S1+S2\right)}}{d_{\left(C1,C2\right)}}}=n\sqrt{\frac{S_{\left(S1\cap S2\right)}/S_{\left(S1+S2\right)}}{\sqrt{{\left(x_{C1}-x_{C2}\right)}^{2}+{\left(y_{C1}-y_{C2}\right)}^{2}}}}$$

where *SC* represents the result of spatial matching rate. *S1*, *S2*, *C1*, *C2* are the areas of the two standard deviation ellipses and their centers of gravity. *x*, *y* are the longitude and latitude of the centers, separately. And *n* is the number of airport points.

### Data collection

The data of COVID-19 cases in this paper collected from the Sina Statistics System for COVID-19 (news.sina.cn/zt_d/yiqing0121) and Wind’s Global COVID-19 Live Update System (wx.wind.com.cn/WindSariWeb/sari/message.html?lan = en). Specifically, the data of COVID-19 cases used in this paper consists of daily data from January 1st, 2020 to November 31, 2022, covering all 54 prefectural level cities in the study area. On this basis, we also summarized the daily data by month, quarter, and year, so as to explored its correlation relationship with air transportation during the calculation and analysis process. It should be noted that there is a lack of the case data of COVID-19 for December 2022 because above systems were no longer updated, so only the case data of October and November will be used as the situation of the 4th quarter in 2022.

The airfield area class and location of airports in Southwest China were from China Civil Airports Network (CCAN), while the transportation volume data were from Southwest China Airport Production Statistics System (info.swcaac.gov.cn/sctj/). The time span of the air transportation data is 2019–2022, which is monthly data. It should be noted that although our research period was during the epidemic of COVID-19, we chose the data of air transportation in 2019 as the "normal conditions" for comparison. In addition, including quarterly and annual data summaries was also carried out.

Socio economic statistics data were mainly obtained from Statistical Yearbooks and Statistical Bulletins of relevant provinces, which are same to the research period in this paper.

## Analysis of results

### The spatiotemporal evolution of the COVID-19 and the air transportation

In terms of the development of the COVID-19 pandemic, as of November 2022, a total of 22,500 cases have been confirmed in Southwest China. Among them, the earliest detected in Chongqing (January 21th, 2020), followed by Chengdu and other places (January 23th, 2020), as of the end of January 2020, cases were found in all provinces in Southwest China but mainly in Chongqing and Sichuan, with only one case in Xizang. After August 2020, the total number of cases in Sichuan exceeded that of Chongqing, and continued to increase significantly. Yunnan Province experienced more than 5 round local epidemic spread in 2021 because of cross-border trade that was so hard to control. As for Xizang, the local epidemic was grim since July 2022, with the number of cases surging and surpassing that of Yunnan and Guizhou. In Guizhou Province, the number of cases has continued to be relatively stable since February 2020, with the lowest overall incidence in the southwestern provinces and autonomous regions. The above situation indicated that the epidemic was related to population size, as well as its socio-economic functions such as tourism industry or cross-border trade. And they all need transportation as a medium for their spatial flow.

From the rank-size situation, the number of cases in the high rank and low rank regions in each quarter was significantly lower than the fitted value. This finding indicated that the changes in the number of cases in various regions were relatively stable. Besides, a large number of central regions contributed to the upward-convexity characteristic of the curve. Combined with the coefficient of variation, the variability in the number of cases across regions was accompanied by a weakening volatility trend. In the first 3 quarters of 2020, the variability was weakened with the spread of cases in different regions; thereafter, by the first quarter of 2022, the number of cases was slightly polarized to some extent, which led to an increase in the variability, and then in the second quarter of 2022, the development of the epidemic was at a low level of dissemination, which led to a significant decrease in the variability; after July 2022, with the rapid development of the epidemic from a few regions to the overall spread, the difference experienced a significant change from peak to minimum.

**Fig 1:**
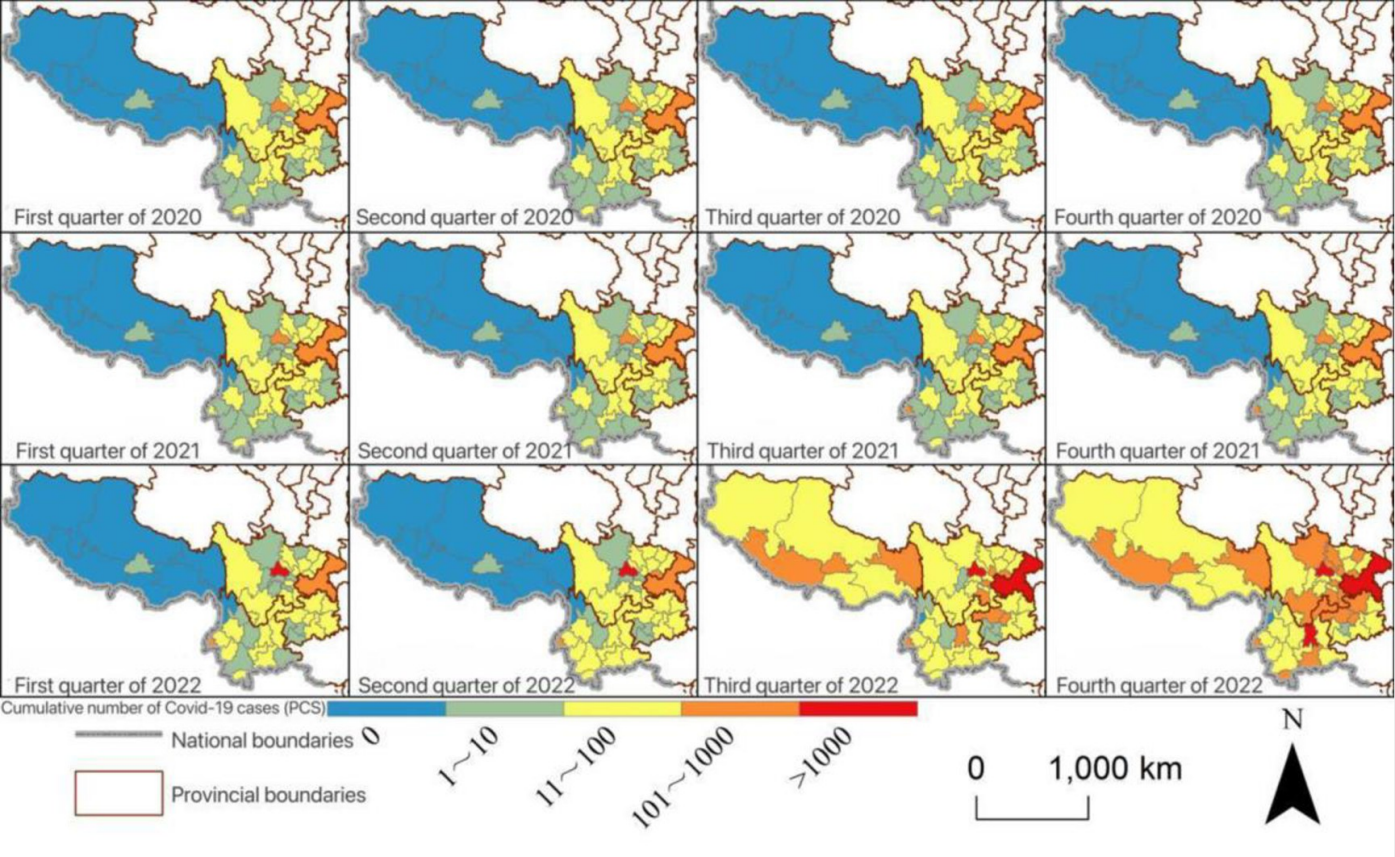
Cumulative number of COVID-19 cases by quarter in Southwest China. The basic map came from the official website of Standard map of China at http://bzdtchinnr.gov.cn/. The drawing approval number is GS (2022) 4313. The data used was calculated by the author.

Spatially, the development of the epidemic was mainly more severe in the regional center cities of Chongqing, Chengdu, and Kunming, and the growth of cases in border cities and provincial border areas such as Dehong was also faster, and since July 2022, Tibet, where the number of cases was previously relatively small, also experienced a significant growth, which caused the epidemic to show a certain degree of westward spreading trend in the spatial distribution (Fig 1). In addition, the results of Moran’s *I* showed that the number of cases across the Southwest China showed negative spatial auto-correlation and tended to be obvious, indicating to a certain extent that the cross-location development of the epidemic in the Southwest China did not take neighborhood diffusion as the absolute main type. The negative spatial autocorrelation characteristics may indicate to some extent that, based on the hypothesis that transportation has an interactive impact on the development of the epidemic, ground transportation may not be closely related to the spread of COVID-19 cases.

**Fig 2:**
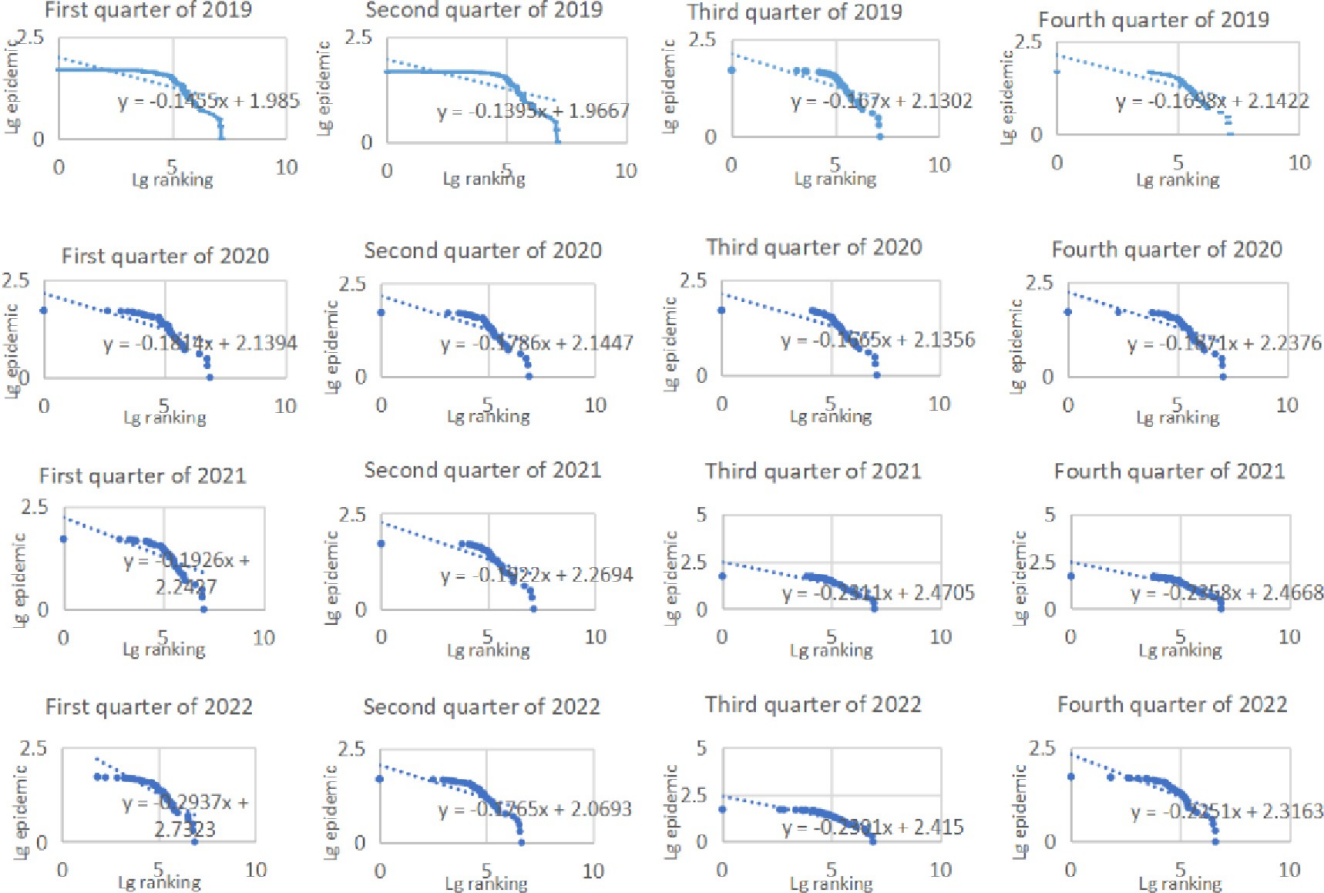
Rank-size distribution of passenger volume in Southwest China.

**Fig 3:**
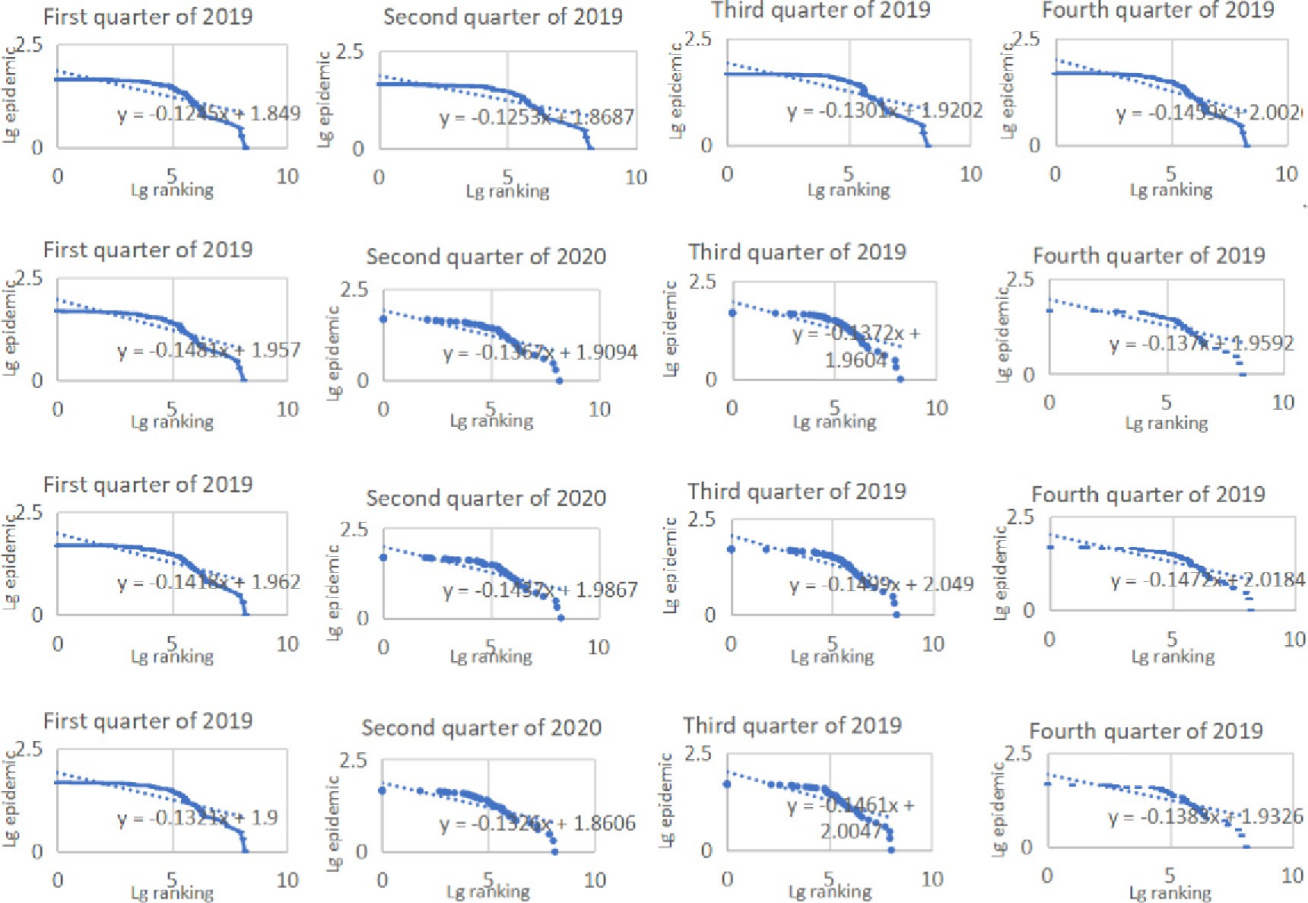
Rank-size distribution of freight volume in Southwest China.

In terms of air transportation, passenger volume declined significantly and showed fluctuations, while freight volume showed the increment tendency. Besides, main trunk airports still occupying the dominant position in the scale of air transportation volume. In the first quarter of 2020, passenger volume at all airports declined significantly compared with the same period in 2019. The fluctuations in different quarters in 2021 were still obvious, while passenger volume in 2022 was the lowest during the study period. Higher-grade airports in central cities such as Chengdu Shuangliu Airport, Chongqing Jiangbei Airport and Kunming Changshui Airport were more affected but also more resilient. However, in combination with the rate of change (excluding the abnormal changes due to the opening and closing of airports), Tongren Fenghuang Airport and Kali Huangping Airport and other feeder airports also had a higher rate of change, which also contributed to the increasing imbalanced distribution in the size and structure of the Southwest China airport system (Fig 2). In terms of freight volume, there was also a sharp decline in the first quarter of 2020. The disparity among various airports was significant, but overall, the freight volume of most airports has increased. Chengdu Shuangliu Airport, Chongqing Jiangbei Airport, and Kunming Changshui Airport still occupied the central position of Southwest China’s air freight traffic scale (Fig 3).

**Fig 4:**
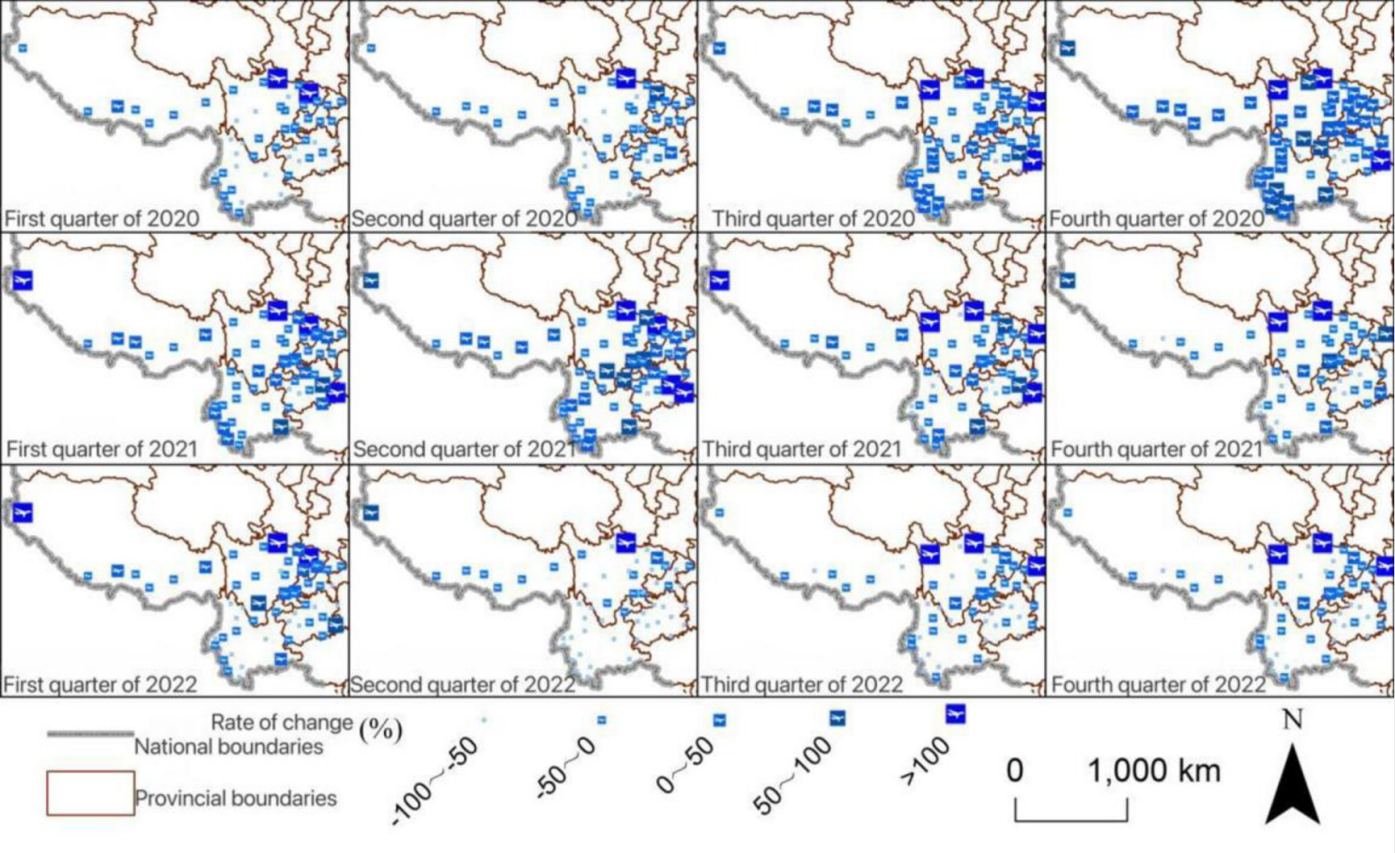
Change rate of freight volume of each airport in Southwest China. The basic map came from the official website of Standard map of China at http://bzdt.ch.mnr.gov.cn/. The drawing approval number is GS (2022)4313. The data used was calculated by the author.

**Fig 5:**
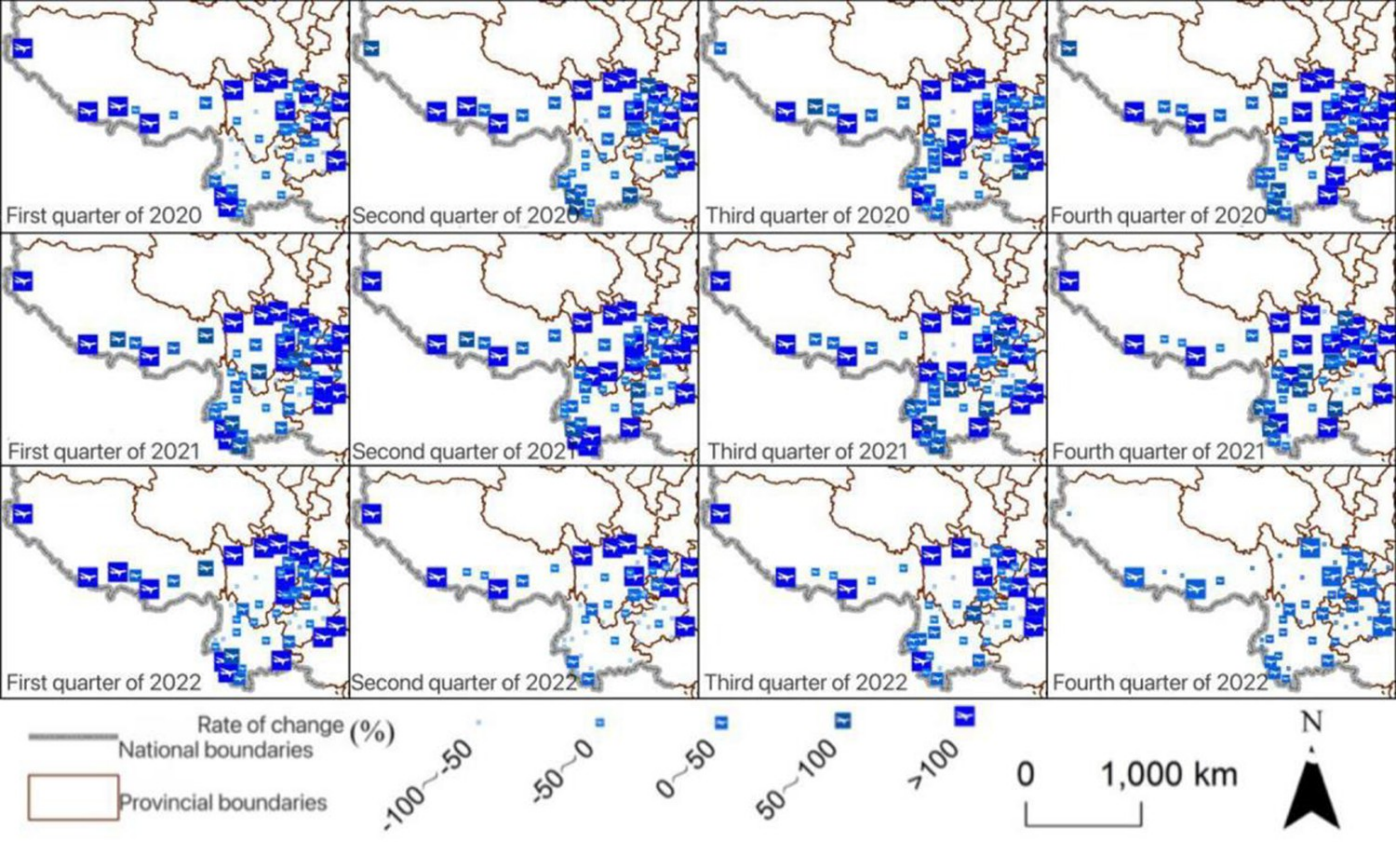
Change rate of passenger volume of each airport in Southwest China. The basic map came from the official website of Standard map of China at http://bzdt.ch.mnr.gov.cn/. The drawing approval number is GS (2022) 4313. The data used was calculated by the author.

Spatially speaking, airports with the significant decline in passenger volume were scattered in the central part of Southwest China. Many airports located in the western part of Southwest China changed slightly in passenger volume. The main trunk airports had also seen a significant rebound in passenger volume (Fig 4). In terms of freight volume in the first quarter, the overall growth rate of eastern airports was once again significant, while after the second quarter of 2022, the growth rate of freight volume at central and southern airports was relatively lower (Fig 5).

### The spatiotemporal relationship between COVID-19 and air transportation volume

During the study period, passenger and freight volume of air transportation in Southwest China declined sharply compare to that in 2019. Although there had been a gradual recovery since the second half of 2020, and it has been relatively stable throughout 2021 when the transport volume gradually recovered to the level of early 2019. However, after March 2022, with the widespread of the COVID-19 in Southwest China, air transportation has once again been affected. The overall decrease in passenger volume was more significant, while the freight volume remained stable. Overall, although the changes during this period were very complex and diverse, the development of the COVID-19 epidemic always brought fluctuations in the scale of air transportation.

Furthermore, based on Pearson correlation coefficient (Table 1), during the study period, it was found that there was a significant positive correlation between the number of newly confirmed COVID-19 cases and the passenger or freight volume. Specifically, from the relationship between COVID-19 cases and the change rate of passenger volume, there was a negative correlation in 2020 and 2022. That is, in areas with more cases, the impacts on air transportation were more significant. In contrast, the negative correlation between the number of cases and the change rate of freight volume was not significant. Overall, under the impact of the epidemic, passenger volume was more affected.

**Table 1:** Test results of correlation between the number of cases and air transportation volume.

	Newly confirmed COVID-19 cases	Passenger volume	Freight volume
Newly confirmed COVID-19 cases	1(0.000***)	0.341(0.001***)	0.401(0.000***)
Passenger volume	0.341(0.001***)	1(0.000***)	0.96(0.000***)
Freight volume	0.401(0.000***)	0.96(0.000***)	1(0.000***)

Note

*** represents that the results can pass the 1% significance level test.

**Fig 6:**
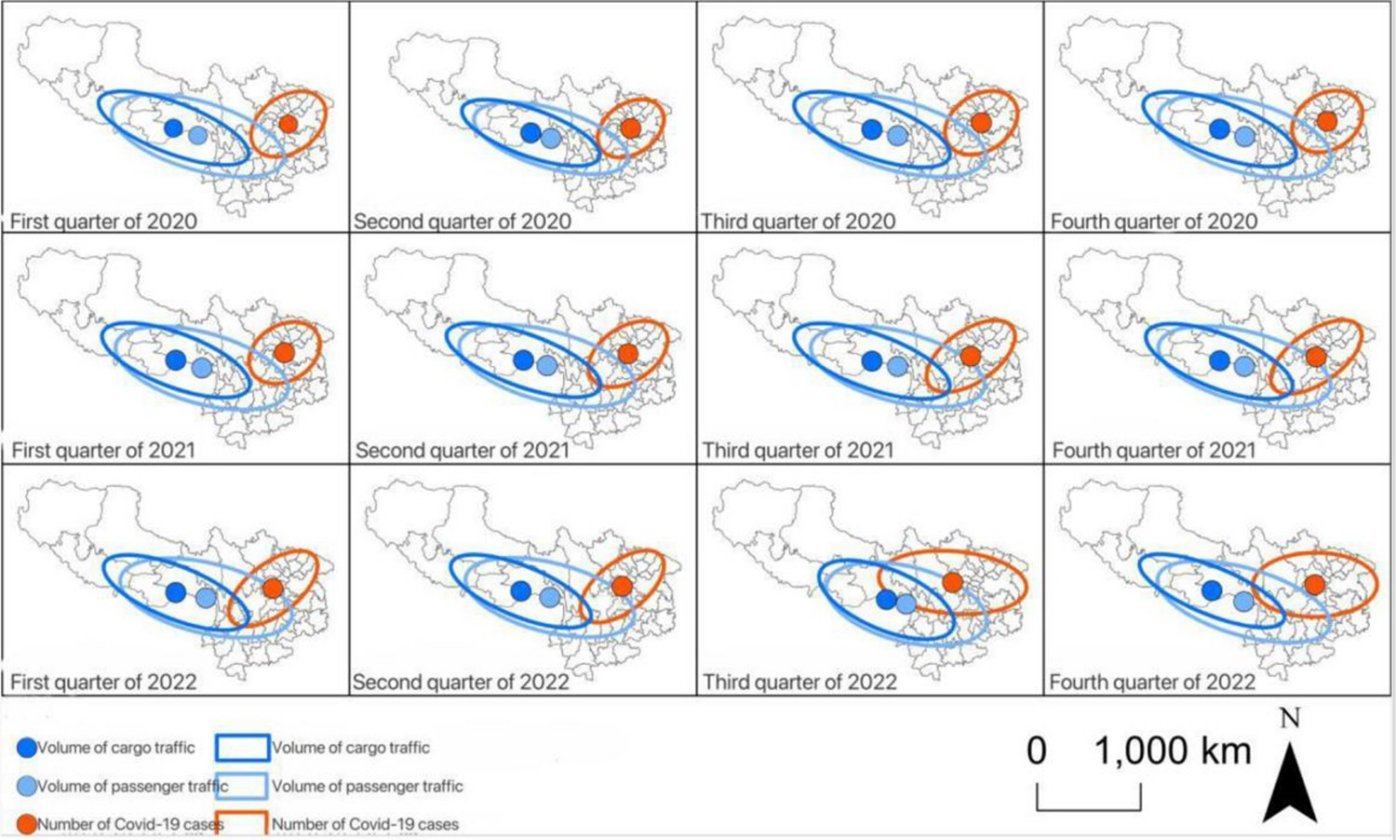
The standard deviation ellipse and the center of gravity of the cases and the distribution of passenger and freight volume of the airport in Southwest China. The basic map came from the official website of Standard map of China at http://bzdt.ch.mnr.gov.cn/. The drawing approval number is GS (2022) 4313. The data used was calculated by the author.

**Fig 7:**
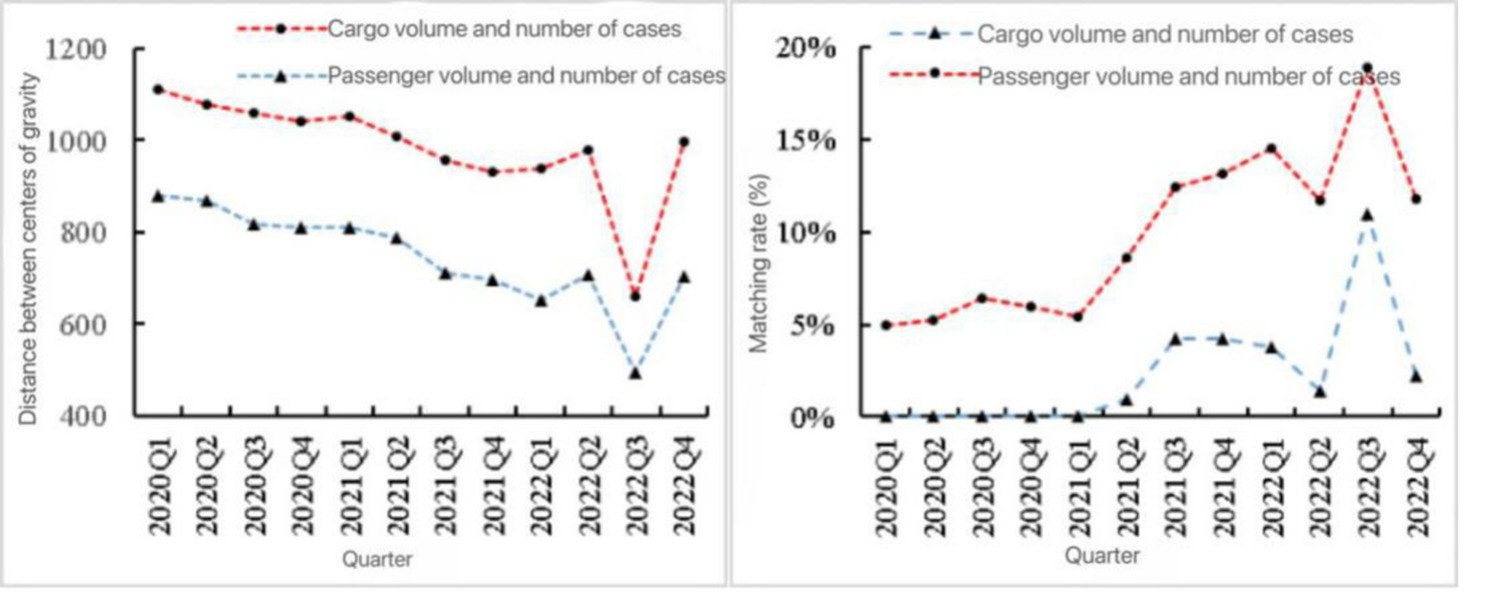
Distance between centers of gravity and spatial matching rates of passenger and freight volume and case number in different seasons in Southwest China.

Then, by the spatial matching index based on standard deviation ellipse and spatial mean center, the quarterly spatial characteristics of the cases and passenger and freight volume in Southwest China from 2020 to 2022 were depicted. First of all, the center of gravity of the number of cases was located in the west of Chongqing in 2020, and gradually shifted westward to Sichuan Province in 2021–2022. In the third and fourth quarters of 2022, the overall distribution changed from southwest to northeast and from east to west, and the distribution of cases expanded, while the center of gravity of passenger and freight volume was located in the Tibet Autonomous Region, and the overall distribution was from northwest to southeast. The results showed that during the study period, the consistency between the number of cases and the spatial distribution of passenger and freight volume in Southwest China gradually increased (Fig 6).Secondly, from the perspective of distance between centers of gravity and spatial matching rate, the variation trend of spatial dislocation degree of passenger volume and freight volume was similar (Fig 7), and both showed a fluctuating downward trend before the third quarter of 2022.It then felt sharply in the third quarter and returned to the level before the drop in the fourth quarter, in which the centers of gravity of passenger and case numbers were consistently closer together, and the distribution of the passenger and case numbers from 2020–2022 was more consistent in scope, while the distribution of the freight volume and case numbers was more consistent in scope. The range consistency between passenger volume and the number of cases was high, while the range consistency between freight volume and the number of cases was relatively weak.

## Conclusion and discussion

### Conclusion

Under the COVID-19 pandemic, there had been significant changes in air transportation. This paper explored the relationship, especially the spatial matching situations, between COVID-19 cases and air transport volume by monthly, quarterly, annual data, as well as methods such as standard deviation ellipse and spatial matching index. Main findings are as follows: Firstly, during 2020–2022, there was a positive relationship between passenger volume and epidemic development, while freight volume increased for most airports in the first quarter of 2020–2022, particularly in the eastern region; Secondly, from the perspective of changes in air transportation volume under the development of the COVID-19, among various types of airports, the changes in transportation volume of main trunk airports were more significant than those of regional feeder airports in remote areas; However, under the influence of the epidemic, main trunk airports still exhibited stronger attraction in passenger volume. That is to say, the passengers who chose to travel by air still tended to choose the main trunk airports and formed the agglomeration distribution pattern which around high-level airports in the provincial capital. Whereas the freight volume had a tendency of equalization among airports in Southwest China; Finally, the consistency of the spatial distribution of the number of cases and the passenger or freight volume in southwest China gradually increased. Among them, the spatial matching rate of the passenger volume and the number of COVID-19 cases was always higher than that of the cases and freight volume, which might indicate that there was a potential but stronger correlation relationship.

### Discussion

In the post-epidemic era, an increase in flights and a recovery in passenger flows became an inevitable trend, especially in tourist cities and main airports. However, the stability of the regional airport system and its service functions should be enhanced.

On this basis, the corresponding measures include optimizing the multi-center airport system, moderately building international airlines, improving the ability to deal with emergency incidents especially for main airports and those in tourist cities. Besides, the capacity of general airports can also be built and strengthened to better carry out emergency rescue and assistance works.

It is also worth noting that, though the impact of epidemic brought by COVID-19 has gradually weakened since the end of 2022, there are still many possible emergencies and public health events may have a significant impact on the ordinary operation of air transportation. Therefore, the relative discussion is still of practical and theoretical significance. However, limited to data collection, this paper has not discussed the specific information on route direction and flights, nor carried out the in-depth discussion on the influencing factors. The above shortcomings can be further focused on in the future, so as to provide reference for the orderly recovery of civil aviation and regional development.
