# Correction: Study on the temporal and spatial relationship between public health events and the development of air transport scale: A case of the Southwest China

**DOI:** 10.1371/journal.pone.0305182

**Published:** 2024-06-07

**Authors:** Zihan Li, Xiwen Deng, Yi Miao, Jinglong Duan

The third author’s name is spelled incorrectly. The correct name is: Yi Miao.

The affiliation 1 for all the authors is incorrect. The correct affiliation 1 is: College of Geography and Environment, Shandong Normal University, Jinan, Shandong, China.

The correct citation is: Li Z, Deng X, Miao Y, Duan J (2024) Study on the temporal and spatial relationship between public health events and the development of air transport scale: A case of the Southwest China. PLoS ONE 19(4): e0301461. https://doi.org/10.1371/journal.pone.0301461.
